# Self-organized criticality as a framework for consciousness: A review study

**DOI:** 10.3389/fpsyg.2022.911620

**Published:** 2022-07-15

**Authors:** Nike Walter, Thilo Hinterberger

**Affiliations:** Section of Applied Consciousness Sciences, Department of Psychosomatic Medicine, University Hospital of Regensburg, Regensburg, Germany

**Keywords:** self-organized criticality (theory), neurodynamical model, theories of consciousness, complexity, phase transition

## Abstract

**Objective:**

No current model of consciousness is univocally accepted on either theoretical or empirical grounds, and the need for a solid unifying framework is evident. Special attention has been given to the premise that self-organized criticality (SOC) is a fundamental property of neural system. SOC provides a competitive model to describe the physical mechanisms underlying spontaneous brain activity, and thus, critical dynamics were proposed as general gauges of information processing representing a strong candidate for a surrogate measure of consciousness. As SOC could be a neurodynamical framework, which may be able to bring together existing theories and experimental evidence, the purpose of this work was to provide a comprehensive overview of progress of research on SOC in association with consciousness.

**Methods:**

A comprehensive search of publications on consciousness and SOC published between 1998 and 2021 was conducted. The Web of Science database was searched, and annual number of publications and citations, type of articles, and applied methods were determined.

**Results:**

A total of 71 publications were identified. The annual number of citations steadily increased over the years. Original articles comprised 50.7% and reviews/theoretical articles 43.6%. Sixteen studies reported on human data and in seven studies data were recorded in animals. Computational models were utilized in *n* = 12 studies. EcoG data were assessed in *n* = 4 articles, fMRI in *n* = 4 studies, and EEG/MEG in *n* = 10 studies. Notably, different analytical tools were applied in the EEG/MEG studies to assess a surrogate measure of criticality such as the detrended fluctuation analysis, the pair correlation function, parameters from the neuronal avalanche analysis and the spectral exponent.

**Conclusion:**

Recent studies pointed out agreements of critical dynamics with the current most influencing theories in the field of consciousness research, the global workspace theory and the integrated information theory. Thus, the framework of SOC as a neurodynamical parameter for consciousness seems promising. However, identified experimental work was small in numbers, and a heterogeneity of applied analytical tools as a surrogate measure of criticality was observable, which limits the generalizability of findings.

## Introduction

Consciousness has been fascinated humankind since its very beginning and the relationship between the aware perception of stimuli and the orchestration of billions of neurons in the brain still is a baffling topic for many researchers all over the world (Melloni et al., 2021). Over the years an abundance of conceptual proposals with distinct philosophical foundations were published and straightforward comparisons can be challenging (Seth et al., 2006). Especially, the conceptual nonlinear dynamical system framework in neuroscience has produced a variety of approaches and hypotheses on the relation between dynamics of neural activity and conscious experience (Cosmelli et al., 2007; Werner, 2007b). Such notions are appealing considering that consciousness reflects an intrinsically dynamical phenomenon, the propagation of information, a temporal process in its nature, notably described as the “stream of consciousness” (James, 1950). Importantly, no current model of consciousness is univocally accepted on either theoretical or empirical grounds and the need for a solid unifying framework is evident (Melloni et al., 2021; Signorelli et al., 2021). One strong candidate for this task is self-organized criticality (SOC) providing an established and competitive model to describe the physical mechanisms underlying spontaneous brain activity and hence, in extension, cognition, behavior and consciousness (Cocchi et al., 2017).

Therefore, the purpose of this work was (i) to review the literature including annual number of publications and citations, type of articles, and applied methods, as well as to outline evidence bringing together theories and experimental evidence on how SOC may account for conscious perception and (ii) to give a conceptual perspective regarding agreements with the most influencing theories on consciousness described in the following.

Theories on how consciousness relates to the physical domain generally start from different premises. Hence, the current two main theories of consciousness, the global workspace theory and the integrated information theory, incorporate distinct approaches to the definition of consciousness (Michel et al., 2018). The global workspace theory (GWT) starts by posing the question of conscious access, i.e., how an external or internal piece of information gains access to conscious processing resulting in a reportable subjective experience. Hence, the central notion behind the GWT is that conscious content is globally available for diverse cognitive (unconscious) processes such as memory and attention, and that consciousness might be a gateway enabling access between otherwise separate neuronal functions (Baars, 2002). Thus, it posits that the function of conscious awareness is the broadcasting of information in the brain and it postulates that information becomes conscious by the activation of long-distance connectivity of “workspace neurons,” which can make the information available to other modular cerebral networks processing information in an unconscious manner (Baars, 1993; Dehaene, 2001). Whereas related stances such as the idea of a “Dynamic Core” proposed by Edelman and Tononi hypothesized thalamocortical and corticocortical reentry as the basic mechanism facilitating the interaction among distant regions of the brain (Edelman and Tononi, 2001), GWT assumes that a nonlinear network ignition associated with recurrent processing amplifies and sustains a neural representation, which allows the global accesses by local processors (Mashour et al., 2020). The GWT further describes ignition as a sudden activation, which may be triggered by an external stimulus or may occur spontaneously and stochastically at rest (Dehaene and Changeux, 2005). The theory has led to a number of empirical studies applying experimental paradigms such as masking, binocular rivalry, and inattentional blindness to investigate a minimal contrast between conscious and nonconscious (i.e., subliminal or preconscious) stimuli (Dehaene and Changeux, 2011).

Distinguishable to the definition of consciousness in terms of brain-wide information sharing, the integrated information theory (IIT) uses a distinct approach claiming that the existence of consciousness cannot be inferred starting from physical systems (Tononi, 2015). In their work, Tononi et al. derived axioms from phenomenological considerations on essential properties required for the physical substrate of consciousness. Centrally, it is argued that an experience is identical to a conceptual structure that is maximally irreducible intrinsically. Going further, the theory advocates a mathematical measure to evaluate not only the quantity but also the quality of consciousness. Hereby, it is argued that a maximum of intrinsic cause–effect power is fundamental, proposing a parameter termed *ϕ*, which reflects the amount of causally effective information that can be integrated across a subset of elements (Tononi, 2004; Oizumi et al., 2014; Tononi et al., 2016; Niikawa, 2020). Due to its level of formalization as a theory, and especially “a calculus to evaluate whether a physical system is conscious” (Tononi and Koch, 2015), the IIT has triggered a lot of responses, debates, and criticisms. Therefore, it has been revised continuously over the years (Oizumi et al., 2014; Cerullo, 2015; Barrett and Mediano, 2019; Kleiner and Hoel, 2021; Kleiner and Tull, 2021).

Moreover, special attention has been given to the hypothesis that neural dynamics might be governed by the phenomenon of SOC. SOC, in the sense of statistical physics, is defined as a specific type of behavior, seen when a system undergoes a phase transition. During a phase transition, macroscopic properties of the system, termed the order parameters, change as a function of a so-called control parameter. For example, when water gets boiled, a phase transition from liquid to a vaporous phase occurs. Here, the order parameter would reflect the phase’s entropy (such as water or vapor), whereas the control parameter is the temperature. Modifying the control parameter gradually changes the order parameter until a specific point, at which the values of the order parameter vary abruptly. Graphically, phase transitions are either marked by a discontinuity of the phase diagram (a jump of the order parameter) or by a point of non-differentiability reflected as a sharp corner. The latter is termed a continuous second order phase transition, which allows the system to be poised exactly between two phases. In that case the system is in the critical state, residing between two qualitative distinct types of behavior such as ordered and disorder. A system at criticality is therefore sometimes referred to as on the “edge of chaos.” If the control parameter is below the critical value, the state is called subcritical, whereas values above the critical state result in a supercritical state (Hesse and Gross, 2014; Roli et al., 2018; Zimmern, 2020). Systems in a critical state show complex behavior with inherent characteristics such as scale-invariance, meaning that no scale in time or space dominates the behavioral pattern. This mode is reflected by spatial and temporal correlations and scaling of a power law over several orders of magnitude. Hence, these give rise to self-similar fractal-like structure over many scales (Heiney et al., 2021).

Alan Turing was probably the first one speculating that the brain could be in a critical regime in his seminal paper on the topic of artificial intelligence written in 1950 (Turing, 1950). A decade later advances in explaining the principles of self-organization and nonequilibrium phase transitions such as Herman Haken’s pioneering work on synergetics and Stuart Kauffmann’s investigations paved the way for understanding the brain in terms of a complex system (Haken et al., 1985; Kauffman, 1993; Haken, 2007). Back then, the potential equivalence between neuronal networks and systems exhibiting a phase transition such as cellular automata, binary lattices evolving iteratively, was highlighted (Hopfield, 1982). This so-called “computation at the edge of chaos” (Packard, 1988; Crutchfield and Young, 1990), was in accordance with theories from Per Bak, promoting critical phase transitions as a mechanisms to generate complexity, ubiquitous 1/f noise and the preponderance of fractal structures in nature. In his book “How nature works” he uses the canonical example of a sand pile (Bak, 1996). The sandpile model, which is analogous to a cellular automata, randomly placing chips on a finite grid, describes the process of a random positioning of sand grains on a pile. This results in a slope, which builds up until it reaches a specific, critical threshold value, the transition point. At this point the system is out of balance and from here on, the dropping of more sand grains leads to an avalanche. During an avalanche the site collapses transferring sand into the adjacent site, extending their slope. This dynamic was found to be governed by power laws (Bak and Wiesenfeld, 1987). However, after a rapid increase of publications in this field in the 1990s, interest slowly receded (Hesse and Gross, 2014) and the conjecture of critical brain dynamics has come a long way before it was put to experimental testing ground and revived (Plenz, 2014).

In 2003, Beggs and Plenz hypothesized that the propagation of activity in networks of cortical neurons is describable by equations that govern cascades indicative of a state of SOC. In their study, they recorded spontaneous negative local field potentials (LFP) of mature organotypic cultures and acute slices of rat cortex using a multielectrode array (Beggs and Plenz, 2003). Indeed, the propagation of synchronized LFP followed a power law with a scaling exponent of −3/2 as it would be predicted from a network of globally coupled nonlinear threshold elements (Eurich et al., 2002). The authors termed this new mode of network activity “neuronal avalanches” (Beggs and Plenz, 2003, 2004). Further, *in vivo* experiments confirmed power law statistic and spontaneous activity in form of neuronal avalanches in cats under anesthesia (Petermann et al., 2009; Hahn et al., 2010), in awake monkeys (Petermann et al., 2009; Klaus et al., 2011) and in rats traversing the wake–sleep cycle (Ribeiro et al., 2010). First signatures of criticality in the human brain were reported by Linkenkaer-Hansen et al. (2001), who focused on the temporal fluctuations employing a method called detrended fluctuation analysis and reported scale-free temporal statistics in EEG data (Linkenkaer-Hansen et al., 2001). In 2013, Shriki and colleagues analyzed resting-state brain activity from 124 participants using magnetoencephalography (MEG). Here, large deflections at single MEG sensors were identified and analyzed as cascades. The authors reported that cascade size distribution obeyed power laws with an exponent of −3/2 at timescales where the branching parameter was close to 1. A scaling and coarse graining of the sensor array did not change this relationship (Shriki et al., 2013). Using intracranial depth recordings in humans it was further shown that avalanche distributions follow a power law, whereby these differed between states of vigilance with larger and longer avalanches during rapid eye movement (REM) sleep (Priesemann et al., 2013). Subsequently, spatial critical dynamics were also described in whole brain functional neuroimaging (fMRI) data (Tagliazucchi et al., 2012).

Intriguingly, work on criticality in physical systems suggest that systems in a critical state exhibit optimal computational properties (Cocchi et al., 2017) and it has been shown that critical dynamics in the brain would be equivalently accompanied by functional benefits (Shew and Plenz, 2013). SOC implies a balanced signal propagation, which can have important implications for the dynamics of neural networks. Such balance is based on the likelihood that one spike causes each other neuron to fire and can be captured by the branching parameter *σ*, which is defined as the ratio of descendants, the number of events in a temporal interval *t* and the ancestors, the number of events in the following interval *t* + 1 (Harris, 2002; Beggs, 2007; Shew and Plenz, 2013). Accordingly, experimental evidence suggest that critical dynamics emerge when excitation and inhibition is balanced (Poil et al., 2012; Massobrio et al., 2015b). Importantly, the balance between independence and interdependence among neurons is fundamental for the transmission and processing of information (Kello et al., 2010). Computational advantages of criticality have been demonstrated in neural network models and empirical recordings. For instance, it has been shown that that the dynamic range of a neural network is maximized at a critical point (Kinouchi and Copelli, 2006; Larremore et al., 2011), which has also been suggested by an *in vitro* experiments manipulating cultures pharmacologically close to criticality (Shew et al., 2009) as well *in vivo* recordings from rats (Gautam et al., 2015). Further, optimal information transmission, storage and capacity has been reported in neuronal models at criticality (Bertschinger and Natschläger, 2004; Haldeman and Beggs, 2005; Del Papa et al., 2017), *in vivo* (Shew et al., 2011) and in animal studies (Fagerholm et al., 2016). Importantly, the observed scale-free patterns close to a critical point of a phase transition imply the largest variability and thus, the largest number of configurations and repertoire of possible brain states (Chialvo, 2018). Further, critical dynamics were proposed as general gauges of information processing and features of healthy brain networks (Massobrio et al., 2015a; Zimmern, 2020; Fekete et al., 2021).

However, the criticality in the brain theory has not been unchallenged (Beggs and Timme, 2012; Wilting and Priesemann, 2019; Destexhe and Touboul, 2021). For instance, debates on the significance of power laws have been hold for a long time in diverse areas of research (Reed and Hughes, 2002; Muñoz, 2018) and it remains discussable to what extent the idea of criticality can be generalized to neurobiology (Gisiger, 2001). Importantly, power laws are one of the hallmarks of SOC but not a sufficient condition (Beggs and Timme, 2012). Hence, whereas all critical systems should exhibit 1/f noise, not all 1/f noise is indicative of criticality (Hesse and Gross, 2014; Zimmern, 2020). Also, power laws can emerge through several mechanisms and non-critical systems are also reported to display power law behavior (Mitzenmacher, 2004; Touboul and Destexhe, 2017; Faqeeh et al., 2019). For instance, Friedman and Landsberg reported features of critical dynamics such as power-law distributions of avalanche sizes and durations in a network with hierarchical modular structure even though underlying dynamical processes were not critical (Friedman and Landsberg, 2013). Further models of neural dynamics suggest that diverse neuronal avalanches can coexist simultaneously, although, the network does not operate in a regime at the edge of a phase transition (Martinello et al., 2017). Additionally, it might be the case that power law regimes may coexist with others suggesting metastability (Kelso, 2012) and that it might be possible that brain areas are driven to the critical point separately (Hesse and Gross, 2014).

To summarize, hitherto, no current model of consciousness is univocally accepted on either theoretical or empirical grounds and the need for a solid unifying framework is evident (Melloni et al., 2021; Signorelli et al., 2021). SOC is a strong candidate providing an established and competitive model to describe the physical mechanisms underlying spontaneous brain activity and hence, in extension, cognition, behavior and consciousness (Cocchi et al., 2017). Thus, SOC could be a neurodynamical framework, which may be able to bring together existing theories and experimental evidence (Werner, 2009, 2013; Tagliazucchi, 2017).

Therefore, the aim of this study was to provide a comprehensive overview of progress of research regarding the relationship of SOC and consciousness. For this purpose, a review of the literature including an analysis of citations was conducted and the most influencing publications on the topic as well as applied analytical tools were determined.

## Materials and methods

Data were retrieved from the Web of Science (WoS) electronic database (SCI-Expanded). A search (All-Fields) with the keywords: “self-organized criticality,” OR “critical dynamics,” OR “phase transition,” OR “neuronal avalanches,” AND “consciousness” OR “conscious percept” was performed, Articles published between 01.01.1998 and 31.12.2021 were included. There were no language restrictions applied. The data were extracted in December 2021 from the Web of Science and exported to plain text. The data were then tabulated in a spreadsheet using Microsoft Excel (Microsoft, Redmond, WA, United States). In total *n* = 81 publications were identified. Of all, the full texts were screened to verify that were in the field of neuroscience referring to SOC in the context of brain dynamics. This was not the case in *n* = 10 articles, whereby, the term criticality was used in association with mobile service delivery, behavior in flocks as well as educational and social sciences. Thus, these articles were excluded from the analysis. The criteria evaluated were the year of publication, the total number of citations, number of citations per year, institution, authors, the journal published, subject, type of articles, used methods, and country of origin. To compare the trends of the criticality and consciousness research separately, two additional searches (All-Fields) were performed with the keywords (i) “self-organized criticality” OR “critical dynamics” OR “neuronal avalanche” AND “brain” as well as (ii) “consciousness” OR “conscious percept” AND “brain” OR “neural activity.” In addition, to quantify the growth in the field of SOC and consciousness with respect to the trends of the topics separately, the ratio between the number of citations was normalized by the total number of citations identified with the search (i) and (ii).

## Results

**Figure 1:**
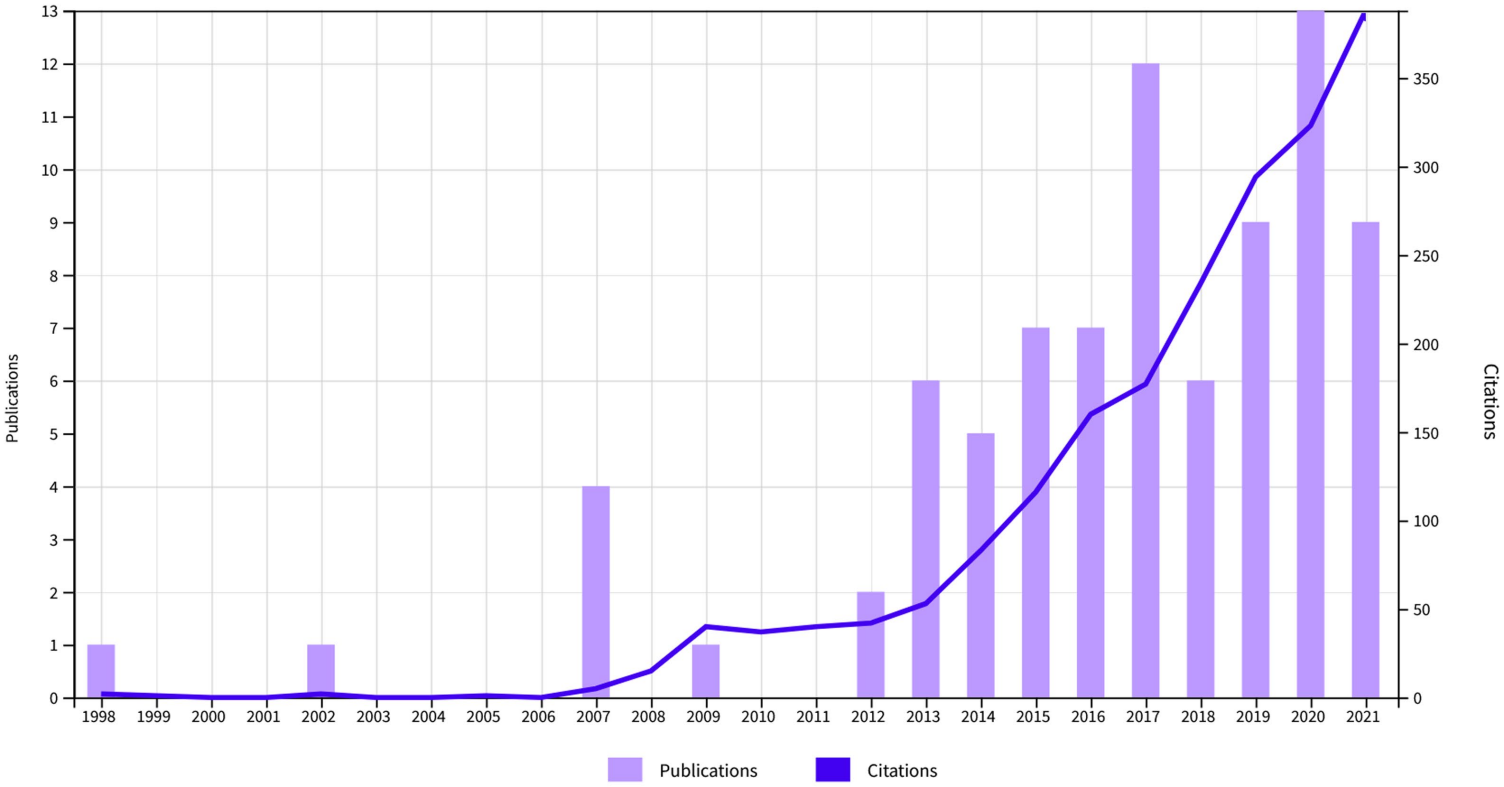
Annual number of publications and citations combining the keywords self-organized criticality (SOC) and consciousness.

**Figure 2:**
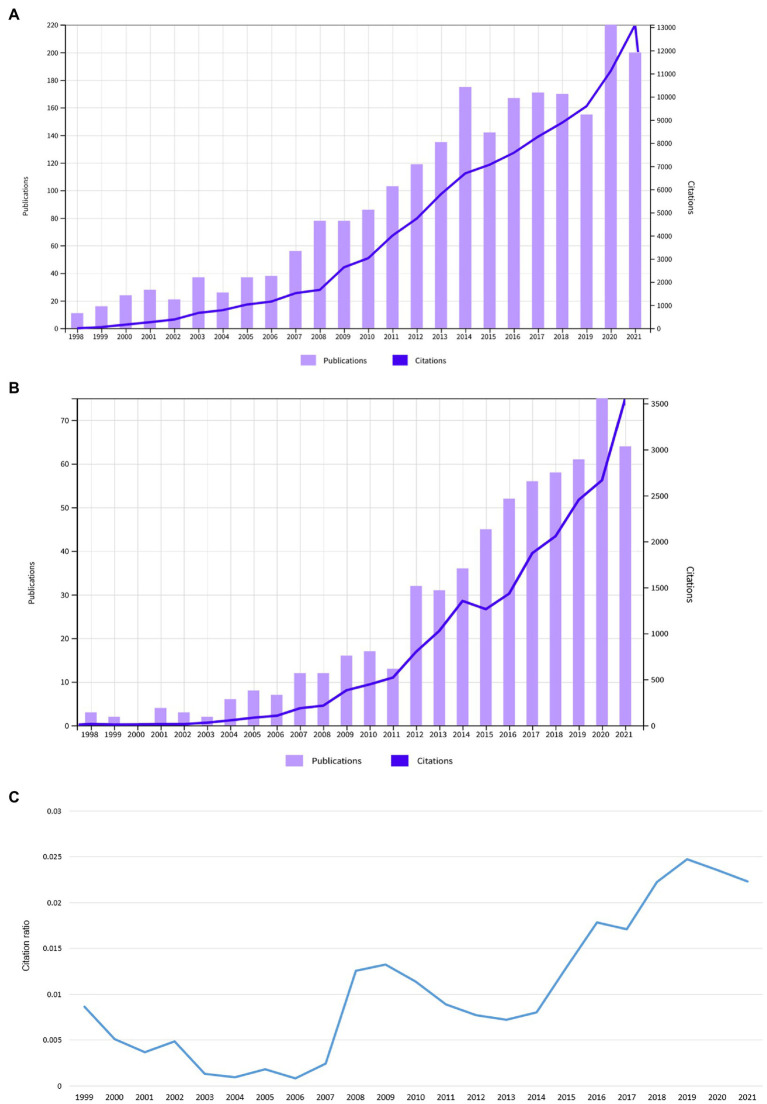
**(A)** Annual number of publications and citations in the field of consciousness research. **(B)** Annual number of publications and citations on SOC in the brain. **(C)** Annual ratio between the number of citations combining the keywords SOC and consciousness normalized by the total number of citations in the field of consciousness research and citations on SOC in the brain.

In total, a number of *n* = 71 publications were included. These had a total number of 1,968 citations (1,878 without self-citations), an h-index of 21, and 24.3 citations averaged per item. Most articles were original (*n* = 36, 50.7%). This was followed by 31 reviews/theoretical articles (43.6%), three proceedings papers (4.2%) and one editorial (1.4%). The annual number of publications was highest in the year 2020 with a local maximum in 2017. The annual number of citations steadily increased over the years (Figure 1). When analyzed independently, both fields showed a growth in articles, citations and the intersection between the two domains thickened (Figure 2).

**Figure 3:**
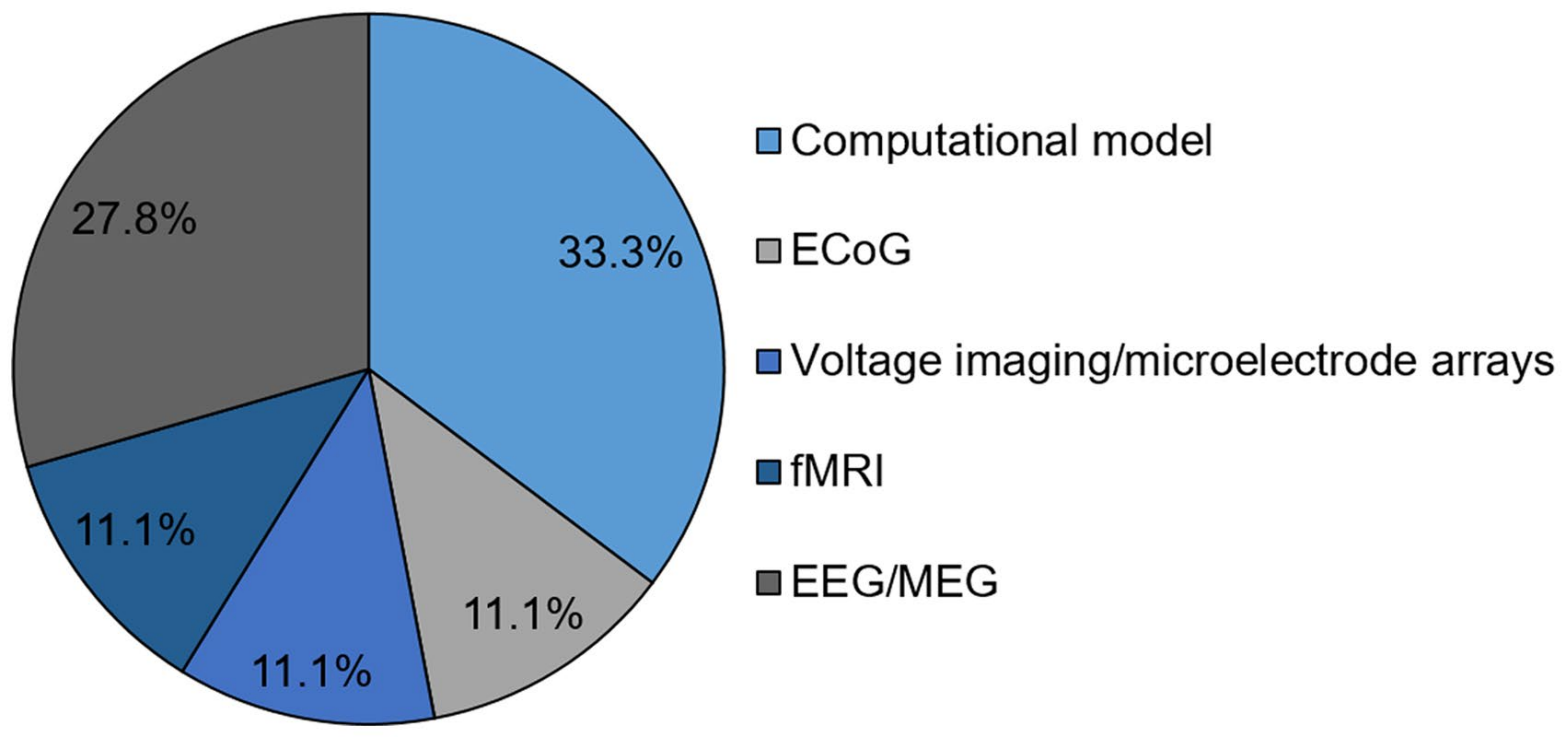
Identified methods of original articles regarding SOC and consciousness given in percentage. EcoG, electrocorticography, EEG, electroencephalography, MEG, magnetoencephalography, fMRI, functional magnetic resonance imaging.

Out of the original articles, *n* = 16 (44.4%) reported on human data and in *n* = 7 (19.4%) studies data was recorded in animals. Regarding the methods, computational models were utilized in *n* = 12 studies (Zare and Grigolini, 2013; Aguilera et al., 2015; Kang et al., 2017; Podobnik et al., 2017; Stramaglia et al., 2017; Abeyasinghe et al., 2018, 2020; Aguilera, 2019; Lee et al., 2019; Avramiea et al., 2020; Kim and Lee, 2020; Popiel et al., 2020), electrocorticography (EcoG) was assessed in *n* = 4 articles (Alonso et al., 2014; Solovey et al., 2015; Krzemiński et al., 2017; Varley et al., 2020b), fMRI in *n* = 4 studies (Liu et al., 2014; Tagliazucchi et al., 2014, 2016; Varley et al., 2020a), voltage imaging/microelectrode arrays in *n* = 4 studies (Fagerholm et al., 2016; Hudetz et al., 2016; Fakhraei et al., 2017; Fekete et al., 2018), EEG/MEG in *n* = 10 studies (Allegrini et al., 2015; Colombo et al., 2016; Irrmischer et al., 2018; Kim and Lee, 2019; Duncan et al., 2020; Dürschmid et al., 2020; Keshmiri, 2020; Fekete et al., 2021; Huels et al., 2021; Kim H. et al., 2021) and EEG and fMRI in one study (Kim M. et al., 2021; Figure 3). Notably, different analytical tools were applied in the EEG/MEG studies to assess a surrogate measure of criticality. These included the detrended fluctuation analysis (*n* = 3), the pair correlation function (*n* = 2), parameters from the neuronal avalanche analysis (*n* = 2), and the spectral exponent (*n* = 1), whereas one study applied multiscale entropy and permutation entropy and another one the autocorrelation function of global alpha oscillations.

In total, 252 authors were identified. The author with the highest number of publications was U. Lee affiliated with the University Michigan, United States, followed by E. Tagliazucchi from Netherlands Institute for Neuroscience. The latter also had the highest number of citations (*n* = 473), followed by G. Werner from the University of Texas with 143 citations from Table 1. There was a significant heterogeneity in the origin of the authors, with most of them coming from the United States (Table 2).

**Table 1:** Top ten authors sorted by the frequency of publications on consciousness and criticality.

Author	Number of articles	Citations
Lee, U.	5 (6.2%)	53
Tagliazucchi, E.	4 (4.9%)	473
Mashour, G.A.	4 (4.9%)	56
Abeyasinghe, P.M.	4 (4.9%)	28
Owen, A.M.	4 (4.9%)	28
Soddu, A.	4 (4.9%)	28
Werner, G.	3 (3.7%)	143
Laurey, S.	3 (3.7%)	69
Allegrini, P.	3 (3.7%)	38
Gemignani, A.	3 (3.7%)	38

**Table 2:** List of top five countries.

Country	Number of articles
USA	36 (44.4%)
Italy	11 (13.6%)
Germany	9 (11.1%)
England	7 (8.6%)
Japan	7 (8.6%)

Articles were published in 51 different journals. The journal Chaos, Solitons and Fractals had the highest number of publications, followed by Neuroimage and Entropy (Table 3). The majority of articles was published as open access (70.4%). The 25 most cited articles on criticality and consciousness were cited between 16 and 323 times (Table 4).

**Table 3:** List of the top ten journals with the most publications and the associated impact factors of the year 2021.

Journal	Record counts	Impact factor
Chaos, Solitons and Fractals	6 (7.4%)	5.9
Neuroimage	6 (7.4%)	6.5
Entropy	5 (6.1%)	2.4
PLOS ONE	4 (4.9%)	3.2
Proceedings of the National Academy of Sciences of the United States of Amerika	2 (2.5%)	11.2
Frontiers in Physiology	2 (2.5%)	4.6
Frontiers in Human Neuroscience	2 (2.5%)	3.2
Frontiers in Systems Neuroscience	2 (2.5%)	3.2
Journal of consciousness studies	2 (2.5%)	1.3
Cultural studies of science education	2 (2.5%)	1.2

**Table 4:** Top 25 most cited publications.

Rank	Title	Total citations	Citations in 2021	Open access	Type of article
1	The entropic brain: a theory of conscious states informed by neuroimaging research with psychedelic drugs (Carhart-Harris et al., 2014)	323	62	Yes	Theoretical
2	Breakdown of long-range temporal dependence in default mode and attention networks during deep sleep (Tagliazucchi et al., 2013)	127	24	No	Original (fMRI)
3	Metastability, criticality and phase transitions in brain and its models (Werner, 2007b)	103	2	No	Theoretical
4	The entropic brain – revisited (Carhart-Harris, 2018)	75	26	No	Theoretical
5	Consciousness as a phenomenon in the operational architectonics of brain organization: Criticality and self-organization considerations (Fingelkurts et al., 2013)	56	4	No	Theoretical
6	Indirect biological measures of consciousness from field studies of brains as dynamical systems (Freeman, 2007)	56	1	No	Theoretical
7	Loss of Consciousness Is Associated with Stabilization of Cortical Activity (Solovey et al., 2015)	55	4	Yes	Original (ECoG)
8	Consciousness as a state of matter (Tegmark, 2015)	45	8	Yes	Theoretical
9	Analysis of Power Laws, Shape Collapses, and Neural Complexity: New Techniques and MATLAB Support *via* the NCC Toolbox (Marshall et al., 2016)	36	7	Yes	Original (methods)
10	The spectral exponent of the resting EEG indexes the presence of consciousness during unresponsiveness induced by propofol, xenon, and ketamine (Colombo et al., 2019)	34	18	Yes	Original (EEG)
11	Cortical Entropy, Mutual Information and Scale-Free Dynamics in Waking Mice (Fagerholm et al., 2016)	30	6	Yes	Original (voltage imaging)
12	Criticality and avalanches in neural networks (Zare and Grigolini, 2013)	29	1	No	Original (network model)
13	Dynamical criticality during induction of anesthesia in human ECoG recordings(Alonso et al., 2014)	27	1	Yes	Original (ECoG)
14	Brain dynamics across levels of organization (Werner, 2007a)	24	1	No	Theoretical
15	Relationship of critical dynamics, functional connectivity, and states of consciousness in large-scale human brain networks (Lee et al., 2019)	23	11	Yes	Original (network model)
16	Random graph theory and neuropercolation for modeling brain oscillations at criticality (Kozma and Puljic, 2015)	23	2	No	Theoretical
17	Scale-Free Functional Connectivity of the Brain Is Maintained in Anesthetized Healthy Participants but Not in Patients with Unresponsive Wakefulness Syndrome (Liu et al., 2014)	23	1	Yes	Original (fMRI)
18	Role of Network Science in the Study of Anesthetic State Transitions (Lee and Mashour, 2018)	22	5	Yes	Theoretical
19	Generalized CNS arousal: An elementary force within the vertebrate nervous system (Calderon et al., 2016)	22	6	Yes	Theoretical
20	Self-organized dynamical complexity in human wakefulness and sleep: Different critical brain-activity feedback for conscious and unconscious states (Allegrini et al., 2015)	21	5	Yes	Original (EEG)
21	The signatures of conscious access and its phenomenology are consistent with large-scale brain communication at criticality (Tagliazucchi, 2017)	19	4	No	Theoretical
22	Ising model with conserved magnetization on the human connectome: Implications on the relation structure–function in wakefulness and anesthesia (Stramaglia et al., 2017)	19	2	Yes	Original (network model)
23	Revisiting the Quantum Brain Hypothesis: Toward Quantum (Neuro)biology? (Jedlicka, 2017)	18	6	Yes	Theoretical
24	Sleep unconsciousness and breakdown of serial critical intermittency: New vistas on the global workspace (Allegrini et al., 2013)	17	1	No	Theoretical
25	Consciousness viewed in the framework of brain phase space dynamics, criticality, and the Renormalization Group (Werner, 2013)	16	2	Yes	Theoretical

EcoG, electrocorticography; EEG, electroencephalography; fMRI, functional magnetic resonance imaging.

## Discussion

The premise arose that SOC is a fundamental property of neural system and that “all human behaviors, including thoughts, undirected or goal oriented actions, or simply any state of mind, are the outcome of a dynamical system, the brain, at or near a critical state” (Chialvo, 2007). In this study, a review study was carried out to determine the current state of the art on SOC as a framework of consciousness.

An upwarding trend in citations on the topic and a maximum of publications in the year 2020 was observed. The publication with highest number of citations was the entropic brain hypothesis by Carhart-Harris et al. (2014), proposing that the “qualia” or subjective quality of any given conscious state, and specifically the “richness” of its content, can be indexed by a quantitative measure of magnitude of entropy in the information theoretic sense (Carhart-Harris et al., 2014). In their original proposal, entropic states were associated with a supercritical regime and low entropic states with a subcritical regime, whereby it was hypothesized that psychedelics tune the brain closer to the critical point compared to normal waking consciousness. To note, their hypothesis has been unchallenged (Papo, 2016). In their revised version, the critical regime accounts for a range of brain states in the spectrum of normal waking consciousness (Carhart-Harris, 2018). The second most influencing paper was an original study by Tagliazucchi et al. (2013), who measured fMRI data across the human nonrapid eye movement sleep cycle (Tagliazucchi et al., 2013). Their study was published in PNAS, the journal with the highest impact factor among the analyzed journals (Table 3). The paper with the third most citations was a theoretical article by Werner (2007a) crosslinking SOC to a “family resemblance” of models of consciousness (Werner, 2007b) including oscillatory synchrony, coordination dynamics (Kelso, 2012), the Dynamic Core hypothesis (Tononi and Edelman, 1998; Sporns et al., 2000), and the global workspace theory (Baars, 2002).

Whereas 43.6% of the articles were theoretical in their nature, the theory has been increasingly put to a testing ground. However, a recent bibliometric evaluation of consciousness theories showed that approximately 3–9 of the citations were based on testing ground. The authors reported that out of five considered theories (global workspace, higher order, integrated information, local recurrent and quantum theories), the IIT had the highest increase in publication and citation count (Yeung et al., 2022).

To note, findings of critical dynamics in the brain did receive substantial critiques (Wilting and Priesemann, 2019). For instance, the quality of power-law fits to empirical data has been scrutinized by demonstrating that some claims of scale-free dynamics lack statistical significance. Hence, stringent statistical tests have been advocated in the detection of critical dynamics (Muñoz, 2018). Thus, especially the work done by Marshall and colleagues providing a freely available MATLAB toolbox for estimating neural complexity and criticality can be regarded as an important advancement incorporating automated maximum likelihood estimation fitting routines for doubly truncated, discrete power-law distribution as well as an automated method for performing and measuring avalanche shape collapses (Marshall et al., 2016).

Interestingly, also a few studies emerged connecting the IIT with the concept of criticality by investigation the explicit relationship between critical exponents and the amount of integrated information (*Φ*). Here, research on criticality and consciousness is largely informed by network models. For instance, Kim and Lee computed a large-scale human brain network model implementing a Kuramoto model on the scaffold of an anatomically informed human brain network structure constructed from diffusion tensor imaging (Kim and Lee, 2019). Arguing that criticality is associated with heightened susceptibility to external stimuli, the pair correlation function (PCF) was calculated as a surrogate measure for susceptibility this was defined as a parameter for criticality. Going further, the authors proposed a metric for *Φ*, defining integrated information as the effective information of the minimum information partition in a system, i.e., the partition of the system at which information loss caused by partitioning is minimized (Kitazono et al., 2018). Then, the network model was modulated by systematically changing the coupling strength. The authors demonstrated that the *Φ* value was maximized at the point of maximized PCF. Second, they analyzed previously published EEG data recorded from seven healthy participants (Blain-Moraes et al., 2015). During the recordings, sevoflurane, an anesthetic agent, was applied, whereby the concentration was first increased from 0.4 to 0.6 to 0.8% and then gradually decreased. The level of consciousness was assessed as the response rate to verbal commands. In comparison to the anesthetic state, conscious resting states showed higher PCF and *Φ*. The authors concluded that a neural network in a critical regime is a necessary condition for information integration in the human brain (Kim and Lee, 2019). Also, Popiel et al. simulated an Ising model on 159 randomly generated, positive weighted *n* = 5 nodes network, which was tuned to a critical point. The parameter *Φ* was calculated as the effective information of the minimum information partition. The results indicated that subcritical regimes can generate high *Φ* values, whereby values were largest near the critical point. The authors concluded that the system would be most conscious, according to the definition given by the 3rd version of the IIT (Oizumi et al., 2014), in the critical regime (Popiel et al., 2020).

Whereas the global workspace theory and the IIT address distinct aspects of consciousness, the first conscious access closely related to the function of conscious awareness and the latter the phenomenology of consciousness, one notable study combined both in the context of criticality (Tagliazucchi, 2017). Enzo Tagliazucchi constructed an anatomical connectivity network inferred from diffusion tensor imaging data. Their computational model represented a variant of the Greenberg–Hastings cellular automaton of excitable dynamics. Hereby, each node of the network can be either be in an inactive, an active or a refractory state. Thus, the model only comprises two parameters, a threshold T, determining the difficulty of the activity to spread and the probability of transitioning from the refractory to the inactive state. For a given of value of T ($T_{c}$) the model depicts a phase transition. Following the argument that Tononi et al. proposed the complexity as an indirect marker of the level of consciousness, the authors calculated neural complexity with the Lempel–Ziv. Also, the amount of integrated information (*Φ*) was determined as the minimum amount of information that is lost when splitting the system into two-subsystems introduced by Barrett and Seth (2011). Further, metastability, defined as the repertoire of configurations that a system explores throughout its temporal evolution was calculated by quantifying the level of global cohesion of the average time series. Then, to model the effect of backward masking, regions of interest chosen from 998 network nodes were serially activated with different delays between the activations. Here, both activations propagated at a certain$\;T_{c}$, whereby the probability of the second activation percolating through the network increased with the delay. Also, they simulated competing stimuli as in the paradigm of binocular rivalry by modeling the propagation threshold of each region of interest. The results reveal that the stimuli did not simultaneously percolate through the network, which the authors interpreted as a dichotomous access to the global network. Important to note, at the critical point of the model, maximal *Φ* and metastability was observed. This findings led the authors to conclude that the two influential theories GWT and IIT could be compatible and that the criticality hypothesis offers a framework in which experimental predications from both can coexist (Tagliazucchi, 2017).

Thus, summarized, the formulation of a regime within the IIT in which segregation and integration occur simultaneously at its maximum corresponding to the conscious state goes hand in hand with the critical state from a dynamical perspective (Tononi, 2004; Tononi et al., 2016; Chialvo, 2018). Further, the abrupt activation facilitating conscious access as described in the GWT may be attributed to SOC, favoring rapid transitions between different states of the system, supporting the adaptive emergence and disappearance of a global workspace (Werner, 2007a, 2009; Kitzbichler et al., 2011).

The main limitation of this study is that the analysis was solely based on Web of Science. Web of Science covers approximately 34,000 journals with over 75 million records and includes the sub-databases Science Citation Index, Social Sciences Citation Index, Arts and Humanities Citation Index, Conference Proceedings Citation Index, Book Citation Index, and Emerging Sources Citation Index (Birkle et al., 2020). Further, the Thomas Scientific impact factor is based on the Web of Science database (Chadegani et al., 2013). In addition, Web of Science is older than the Scopus database, which has been launched by Elsevier Science in 2004, and provides better graphical rankings of the citation analysis and is more detailed than the citation analysis of Scopus (Falagas et al., 2008). However, Web of Science queries the citation, abstract, and keyword identifiers, but one has to acknowledge that articles mentioning the keywords of interest in the methods sections are omitted, and thus, completeness of the identified list of articles on consciousness and SOC cannot be fully ensured.

The topic is compelling as principles such as SOC describing outcomes of collective phenomena in any complex dynamical system, provide a model suitable to situate the phenomenon of consciousness within universal laws of the physical world (Fingelkurts et al., 2013). Whereas over the years some authors have claimed that consciousness is entirely beyond the reach of science or regard consciousness a fundamental feature in form of a ubiquitous field pervading the universe (Seth, 2010; Keppler and Shani, 2020), the theory offers the possibility of empirically testing mathematical measures as neurophysiological indices for consciousness. Thus, in conclusion, the framework of SOC as a neurodynamical model of consciousness is promising synthesizing the current most influencing theories in the field of consciousness research. However, identified experimental work was small in numbers and a heterogeneity of applied analytical tools as a surrogate measure of criticality was observable, which limits the generalizability of findings. Future studies will reveal whether this issue and current pitfalls such as unprecise terminology, the determination of a biological plausible control parameter and debates on the significance of power laws can be overcome (Beggs and Timme, 2012; Wilting and Priesemann, 2019; Zimmern, 2020).

## Data availability statement

The raw data supporting the conclusions of this article will be made available by the authors, without undue reservation.

## Author contributions

NW conceptualized the study, analyzed the data, and wrote the manuscript. TH supervised the project and revised the final version of the manuscript. All authors discussed the results, contributed to the article, and approved the submitted version.

## Conflict of interest

The authors declare that the research was conducted in the absence of any commercial or financial relationships that could be construed as a potential conflict of interest.

## Publisher’s note

All claims expressed in this article are solely those of the authors and do not necessarily represent those of their affiliated organizations, or those of the publisher, the editors and the reviewers. Any product that may be evaluated in this article, or claim that may be made by its manufacturer, is not guaranteed or endorsed by the publisher.
